# Primary pain generator identification by CT–SPECT in a patient with low back pain: a case report

**DOI:** 10.1186/s13104-017-2458-3

**Published:** 2017-03-21

**Authors:** Gabriel Tender, Adriana Constantinescu, Andrew Conger, Anthony DiGiorgio

**Affiliations:** 1 Department of Neurosurgery, Louisiana State University, 2020 Gravier Street, Suite 744, New Orleans, LA USA; 2 Department of Oncology, University of Craiova, Str. Alexandru Ioan Cuza, 200585 Craiova, Romania

**Keywords:** CT–SPECT, Degenerative spine, Low back pain, Primary pain generator

## Abstract

**Background:**

Chronic low back pain is one of the most common conditions encountered in the middle-age population. Identifying the primary pain generator is notoriously difficult. The computed tomography–single-photon emission computed tomography (CT–SPECT) is emerging as a new diagnostic modality for this purpose.

**Case presentation:**

This 68-year-old Caucasian male presented with intractable low back pain refractory to maximal conservative treatment, including medication and extensive physical therapy. The lumbar computed tomography, magnetic resonance imaging, and flexion–extension X-rays showed advanced degenerative changes throughout the lumbar spine, but no single level significantly worse than the others. The CT–SPECT showed markedly increased uptake at the L1–2 disc level and only minimal uptake at the other levels. The patient underwent a minimally invasive lateral L1–2 fusion with near-complete resolution of his low back pain.

**Conclusions:**

The CT–SPECT may provide a unique tool in establishing the primary pain generator in patients with degenerative spine disease.

## Background

Low back pain (LBP) is the most common cause of disability between the ages of 20 and 45 in the United States [1]. While the initial episodes of LBP are usually transient and respond well to non-steroidal anti-inflammatory medication and physical therapy, the natural progression of degenerative spine disease is towards chronic axial pain, with or without radiculopathy, depending on the degree of associated lateral recess and/or foraminal stenosis. However, it is extremely difficult to pinpoint the primary pain generator in patients with such complex spine pathology. Common investigations include, in increasing order of complexity, flexion–extension X-rays (which may demonstrate instability), CT (which may determine bony abnormalities), and MRI (which may identify disc and/or facet joint pathology, among other things). Other diagnostic tools, such as discography, attempt to recreate the patient’s “usual” pain by injecting the respective disc with contrast media under pressure [2]. Unfortunately, discography has significant drawbacks and may actually accelerate disc degeneration. While these tests may show structural abnormalities, no single test has reliably predicted the primary pain generator.

Single-photon emission computed tomography (SPECT) uses detection of 99mTc-Technetium bound to osteoblasts [3] to gain information on the amount of bone remodeling activity in the spinal axis. Using imaging merging software between the SPECT and CT, we can thus identify with a high degree of anatomic precision which parts of the spine exhibit increased osteoblastic activity. This activity may be indicative of pain generation [4, 5].

We report an illustrative case of a patient whose pain source could not be identified by the traditional imaging techniques. The primary pain generator was identified by CT–SPECT and treated by a minimally invasive spinal fusion, which relieved the pain and thus confirmed the diagnosis.

## Case presentation

A 68-year-old Caucasian man presented with several years history of low back pain, occasionally radiating to the left lower extremity. The pain was described as 10/10 in intensity (on the Visual Analog Scale, with 1 = no pain and 10 = the worst pain ever experienced), incapacitating, improved in supine position and increased by walking or standing. The patient has already tried extensive conservative treatment, including medication (non-steroidal anti-inflammatories such as ibuprofen and naproxen, occasionally oral opioids such as oxycodone/acetaminophen, and muscle relaxants such as cyclobenzaprine) and physical therapy, with minimal relief. His past medical and surgical history was significant for hypertension and depression, and a thoracic 11–12 laminectomy and fusion about 8 years prior to presentation, respectively. The patient had a 15-pack-year history of smoking cigarettes, but had quit smoking 20 years prior to presentation. The neurological examination was normal. Preoperative routine laboratory tests: complete blood count (CBC), basic metabolic panel (BMP), prothrombin time (PT), partial thromboplastin time (PTT), and urinalysis (UA) were normal.

**Fig. 1:**
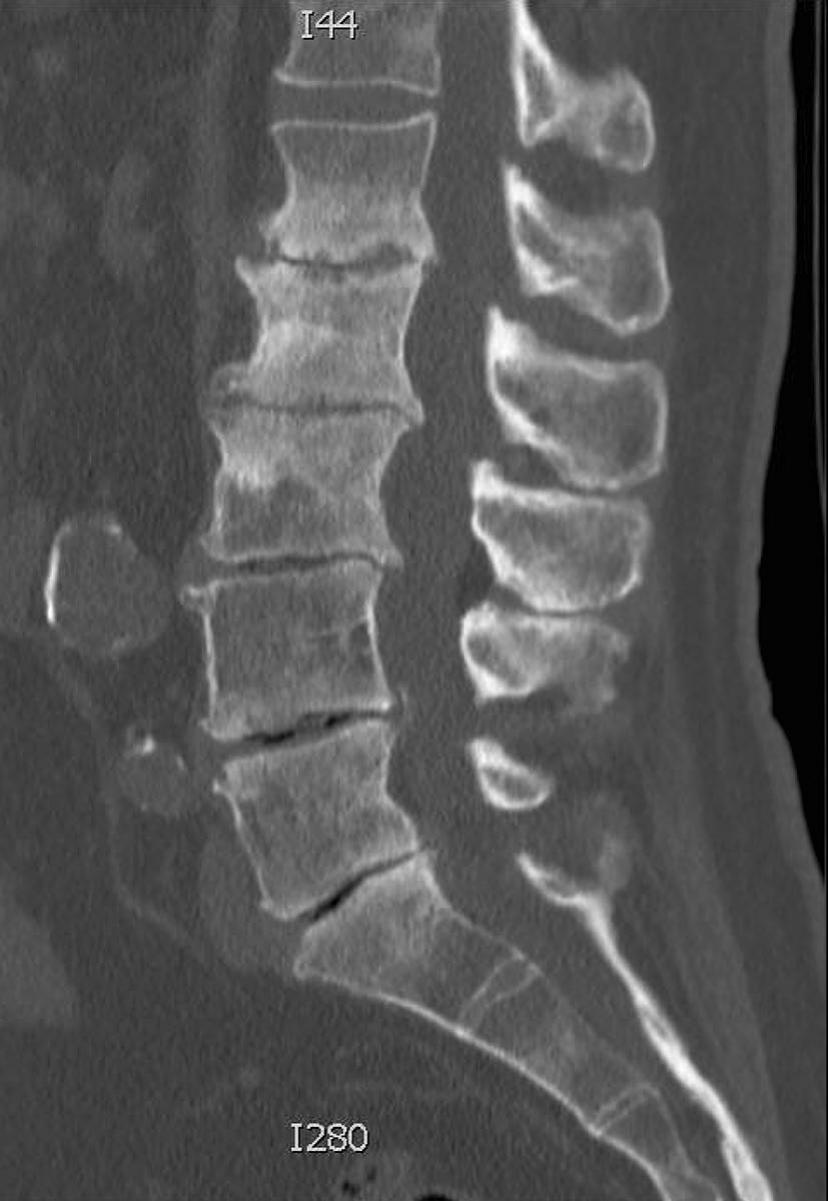
Computed tomography (CT) of the lumbar spine, sagittal view, illustrating the extensive degenerative changes of the lumbar spine. (Technique: Multiple thin cut axial images were acquired through the lumbar spine without contrast. 3D postprocessing and multiplanar reformatting was performed on the source images at the Voxar workstation by the radiologist)

**Fig. 2:**
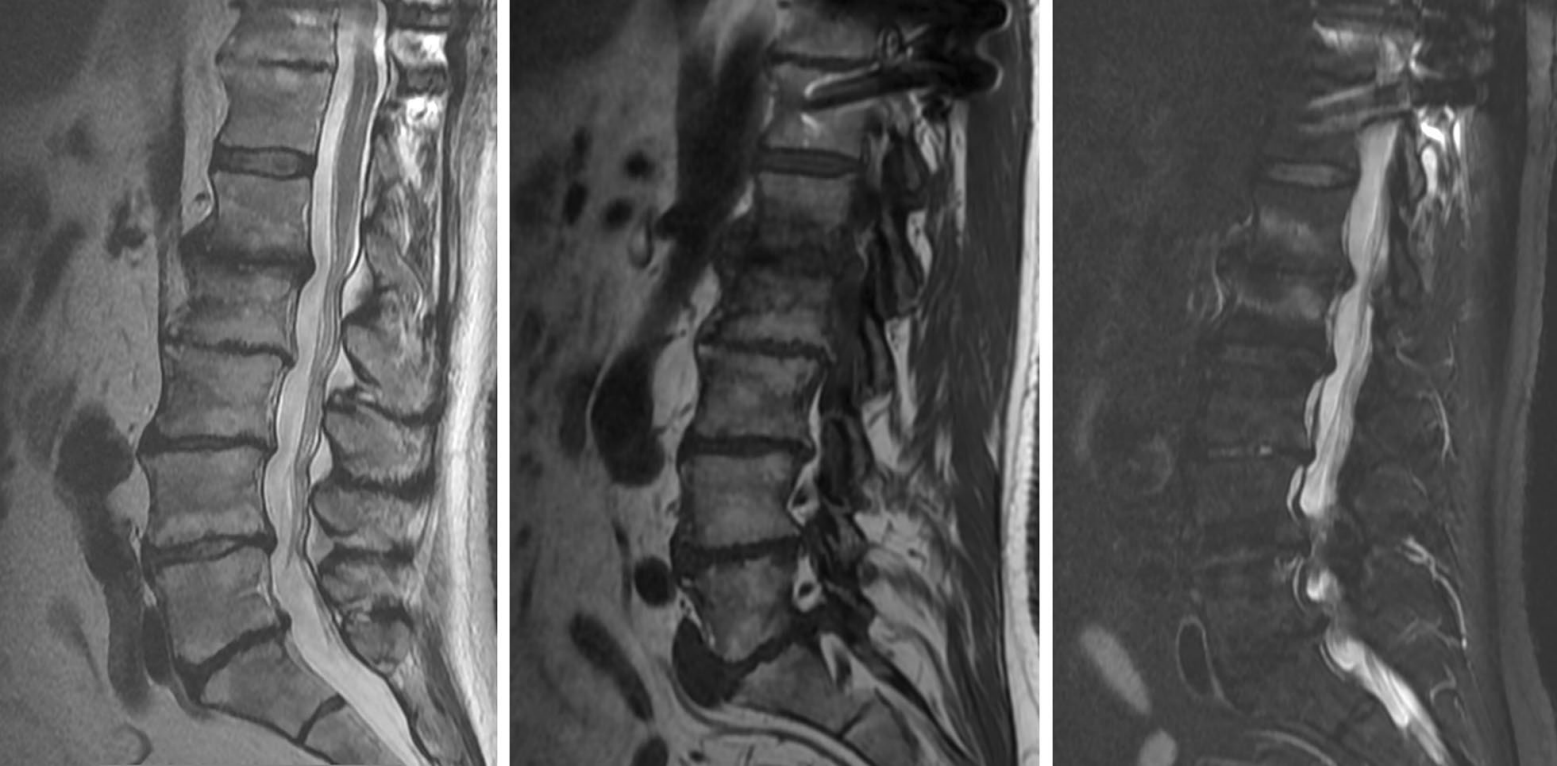
Magnetic resonance imaging (MRI) of the lumbar spine. *Left* sagittal T2-weighted, *center* sagittal T1-weighted, and *right* sagittal T2 with fat suppression images, confirming the advanced degenerative changes, but without any critical central canal stenosis (technique: sagittal images were obtained with fast-spin echo T1-weighting, T2 weighting, and T2 with fat suppression. Axial fast-spin echo T2 weighted images were also obtained)

The flexion–extension and lateral bending lumbar X-rays revealed extensive degenerative changes in the lumbar spine, but no grossly abnormal motion. A lumbar CT confirmed the loss of disc height at all lumbar levels and sclerotic changes, particularly at L1–2 and L2–3 (Fig. 1). The MRI confirmed the CT findings and also showed multi-level foraminal stenosis, but no critical central canal stenosis (Fig. 2).

**Fig. 3:**
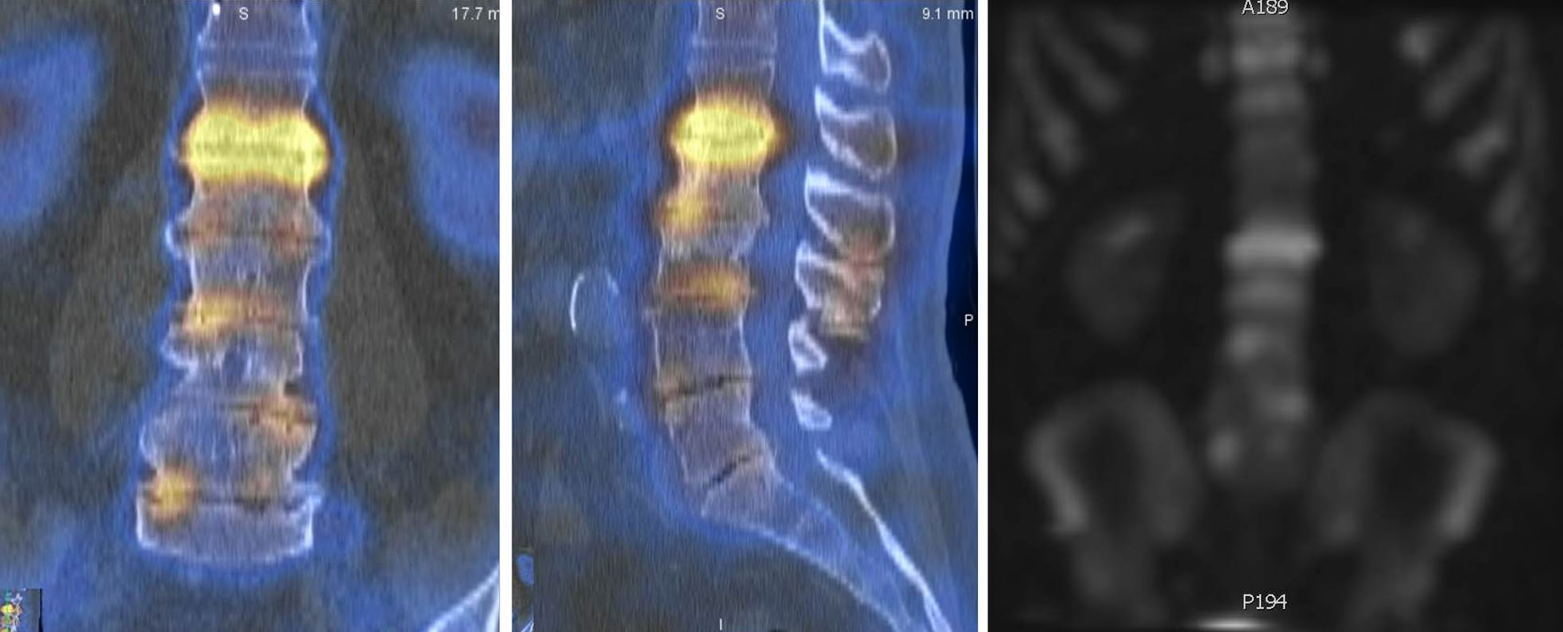
CT–SPECT of the lumbar spine. *Left* coronal and *center* sagittal merged images show increase Technetium uptake at the L1–2 level and minimal uptake at the other levels. *Right* the whole body bone scan (technique: 33.4 mCi of Technetium 99m MDP were administered intravenously. Bone scan images with SPECT imaging was obtained of the lumbar spine. These images were fused to CT lumbar spine of same date)

**Fig. 4:**
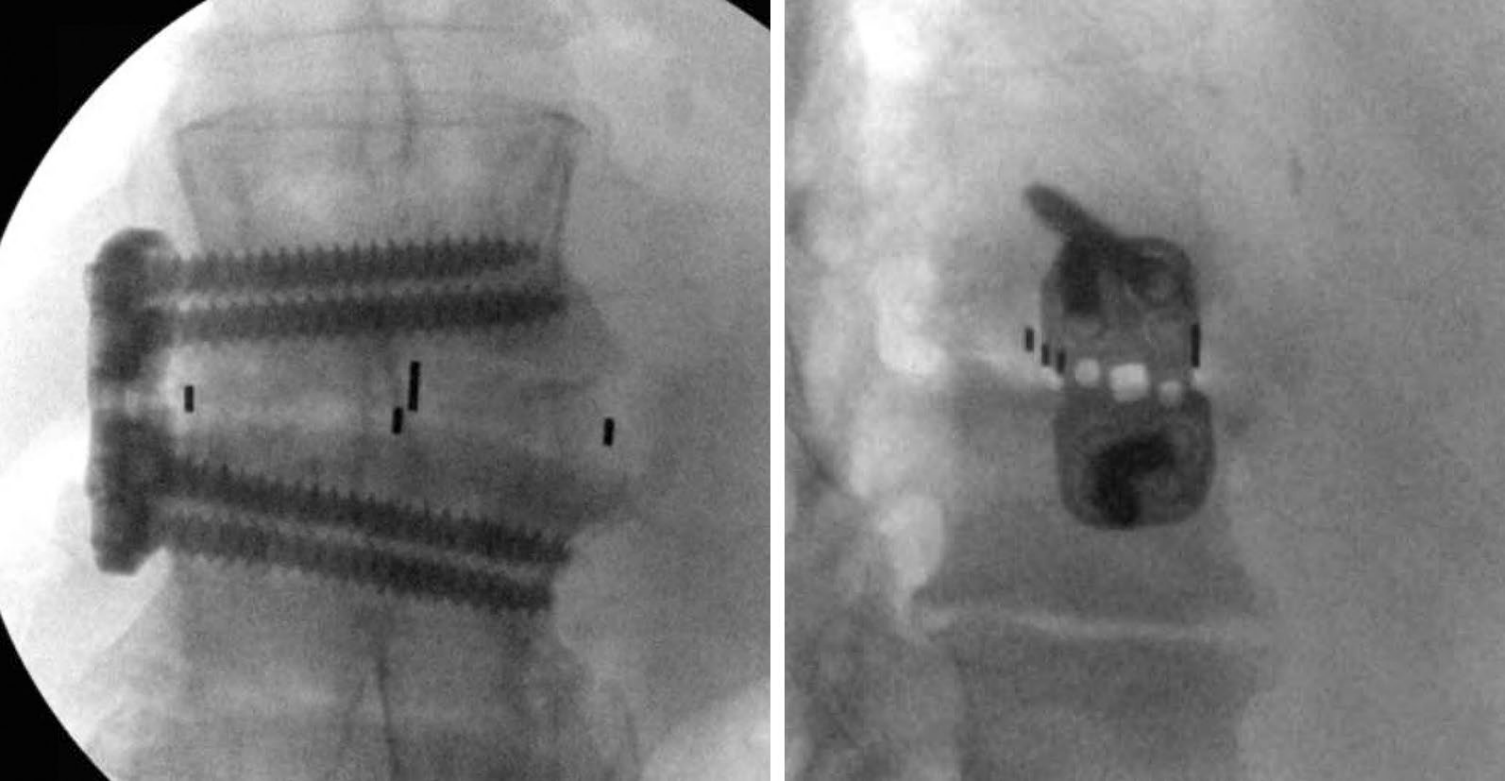
Postoperative X-rays of the lumbar spine. *Left* antero-posterior and *right* lateral images illustrating the L1–2 interbody spacer with a lateral plate

The patient was advised regarding the results of imaging and was told that, based on the available information, we could not reliably identify a single pain generator. A CT–SPECT was obtained and demonstrated significantly increased uptake at the L1–2 disc level, with only minimal uptake at the other degenerated levels (Fig. 3). The patient underwent a lateral retroperitoneal/retropleural approach for a minimally invasive L1–2 interbody fusion with lateral plate (Fig. 4). At the 3-month postoperative visit, the LBP decreased to 1/10 on the average and 2/10 at its’ worst, and these results were maintained at patient’s last clinic visit, 12 months after the surgery. The usage of Percocet^®^ (oxycodone 10 mg + acetaminophen 325 mg) decreased from 3 to 4 per day before the surgery to occasionally one per week at 3-months postoperatively. The rest of medication (aspirin, lovastatin, amlodipine) usage remained the same.

## Discussion

SPECT has been used for several decades to identify bone abnormalities [6]. Recently, clinicians have started to combine the functional value of SPECT with the anatomical accuracy of CT, in order to determine the potential pain generators and treatment targets [7–9].

Identifying the primary pain generator in patients with degenerative spine pathology makes the difference between therapeutic success and failure. Surgical fusions may provide superior results compared to medical treatment in patients with an obvious primary pain generator (e.g., spondylolisthesis) [10]. The situation is different in patients with extensive degenerative spinal changes, with no particular segment significantly worse than the others. In these patients, the traditional options are either conservative treatment or extensive surgery that would address all the levels, but would also incur tremendous risks. We believe that CT–SPECT provides a major diagnostic advantage and may offer these patients a third option. If the CT–SPECT identifies only one or two levels with increased activity, which can be treated by a much smaller operation, we may provide the desired pain relief with minimal associated morbidity.

As previously demonstrated, the SPECT is based on 99mTc-Technetium uptake by the osteoblasts [3]. The study will obviously show increased activity at sites where the bone is attempting to grow, such as fractures or postoperative fusion sites. However, the SPECT will also show increased activity at sites that have abnormal motion, which the body tries to spontaneously fuse, in order to stop the abnormal motion and hence the pain. We are in the process of conducting a prospective study to evaluate the sensitivity and specificity of this test for patients with pain of spinal origin. In the reported case, the CT–SPECT was the only study that could positively identify the primary pain generator, and this was confirmed by the near-complete pain relief after surgical fusion of that segment.

## Conclusions

The CT–SPECT may provide a unique diagnostic advantage over the current modalities in identifying the primary pain generator in patients with pain of degenerative spinal origin.

## Abbreviations

CT–SPECT: computed tomography–single-photon emission computed tomography
CT: computed tomography
MRI: magnetic resonance imaging
LBP: low back pain
CBC: complete blood count
BMP: basic metabolic panel
PT: prothrombin time
PTT: partial thromboplastin time
UA: urinalysis
